# Emotion Processing in Women with Schizophrenia Is Menstrual Cycle Phase and Affective Valence Dependent: An fMRI Study

**DOI:** 10.5402/2012/656274

**Published:** 2012-03-01

**Authors:** Adrianna Mendrek, Josiane Bourque, Annie Dubé, Nadia Lakis, Julie Champagne

**Affiliations:** Centre de Recherche Fernand-Seguin, Department of Psychiatry, Université de Montréal, 7331 Hochelaga, Montreal, QC, Canada H1N 3V2

## Abstract

Despite a large number of functional neuroimaging investigations of emotion processing in schizophrenia, very few have included women. In the present study 21 schizophrenia and 23 healthy women underwent functional MRI (3T) on two occasions (during the follicular and luteal phase of their menstrual cycle) while viewing blocks of emotionally negative, positive and neutral images. During exposure to negatively charged images patients showed relatively less activations than controls during the luteal phase, but no between-group differences were observed during the follicular phase. In contrast, the exposure to positively valenced material produced no significant interaction, but the main effect of group; schizophrenia patients exhibited less activation than healthy controls during both phases of the menstrual cycle. This is the first study demonstrating that atypical neural activations associated with emotion processing in women diagnosed with schizophrenia depend on the menstrual cycle phase and on the affective valence of presented stimuli.

## 1. Introduction

Schizophrenia is a complex and clinically heterogeneous psychiatric disorder with unknown etiology, age at onset in late adolescence/early adulthood, and a lifetime prevalence of approximately 1% [1, 2]. One of the hallmark characteristics of this devastating disorder is a disturbance in emotion processing, which has been demonstrated in numerous behavioral, physiological, and functional neuroimaging investigations that employed tasks ranging from passive viewing of emotional material, through to facial emotion identification and emotional memory [3–8].

Although widely investigated, the neural correlates of atypical emotion processing in schizophrenia patients are still not well understood. For instance, while the majority of studies report diminished activations in patients relative to healthy subjects in several regions implicated in affect (e.g., hippocampus, amygdala, medial prefrontal, orbitofrontal cortex, and cingulate cortices) (e.g., [9–12]), others have found no effect or increased neural reactivity to emotionally charged material (e.g., [13–15]). In our recent study we have observed diminished activations during retrieval of negatively valenced emotional material but enhanced activations during positively valenced condition in clinically stable schizophrenia patients relative to controls [8]. Thus, one important factor to consider is affective valence of presented stimuli. Another important variable is gender of tested individuals, as numerous studies in the general population have demonstrated significant differences between men and women in brain activations during performance of emotional tasks (e.g., [16–20]).

Despite the large number of functional neuroimaging investigations of emotion processing in schizophrenia, very few have included women and, even when they did, the sample sizes were typically too small to allow for examination of brain activations specifically in women. This is not surprising given that the prevalence and incidence of schizophrenia is greater in men than in women during the first half of life (below 40–45 years of age) [21], but this should not prevent us from studying female patients. In one of our previous studies of sex differences in emotion processing in schizophrenia, we observed a different pattern of cerebral activity between male and female patients [22], and we have subsequently found that the symptom profiles correlated differently with brain activations [23]. These results point to the importance of investigating two sexes separately while evaluating emotion processing in psychoses.

The purpose of the present study was to examine brain activations associated with emotion processing in female patients relative to a female comparison group. Given the implication of sex steroid hormones in emotion processing (e.g., [24–26]) and in schizophrenia [27–29], we compared patterns of brain activations during two different stages of the menstrual cycle: follicular (associated with high estradiol and low progesterone levels) and luteal (characterized by a lower estradiol to progesterone ratio).

Based on majority of studies in males with schizophrenia (e.g., [9–12]), we hypothesized that, relative to healthy women, female patients will demonstrate decreased brain activations during emotion processing regardless of the menstrual cycle phase or valence of presented stimuli. Several recent reports in healthy premenopausal women have suggested that elevated levels of estradiol diminish, while progesterone enhances neural responsiveness to emotional situations (particularly highly arousing negative stimulation) [24, 30–32]. Thus, in the present study, we have performed correlational analyses between brain activations and levels of estradiol and progesterone. Consistently with the literature in healthy women, we predicted to find negative correlations between cerebral activations and estradiol, and positive correlations with progesterone (especially during processing of aversive images). In comparison, because there have been some reports of the overall diminished levels of ovarian hormones in women with schizophrenia (e.g., [33–35]), we expected to replicate this finding and to see attenuated relationship between hormones and brain activations in patients relative to healthy women.

## 2. Materials and Methods

### 2.1. Participants

Twenty-one women were meeting DSM-IV criteria for schizophrenia [36], in a stable phase of their illness (defined as no relapse within the last two months and no change in their antipsychotic treatment within the last month), and 23 healthy premenopausal women participated in the study. The groups were matched for age, handedness (Edinburgh Inventory) [37], and parental socioeconomic status (National Occupational Classification; NOC) [38] (Table 1). All participants reported having regular menses (cycles ranging from 25 to 33 days).

All patients were reevaluated by experienced psychiatrists before being assigned to the research group (DSM-IV, criteria A-E); affective, schizoaffective, and schizophreniform psychoses were excluded. Symptom severity was rated according to the positive and negative syndrome scale (PANSS) [39]. Illness onset was defined as the date of the first psychiatric consultation. All the patients received at least one atypical antipsychotic (15 patients received one and 6 received two): clozapine: *n* = 8; mean dosage = 350.00 ± 124.64 mgs; olanzapine: *n* = 6, mean dosage = 15 ± 5.48 mgs; risperidone: *n* = 8, mean dosage = 2.63 ± 1.41 mgs; quetiapine: *n* = 3, mean dosage = 587.50 ± 271.95 mgs; ziprasidone: *n* = 1, dosage = 200 mgs. Control participants were screened with the nonpatients edition of the Clinical Interview for DSM-IV (SCID) [40] to exclude presence of any Axis-I disorders.

General exclusion criteria included age below 18 or above 45 years, lack of menstrual cycle, any past or present neurological disorder, alcoholism or drug abuse, abnormal uncorrected vision, or any contraindication for MRI, such as a cardiac pacemaker, an aneurysm clip, a metal prostheses or cardiac valve replacement, the presence of metal in an eye or any part of the body, certain dental work, or claustrophobia.

In agreement with the Declaration of Helsinki, written informed consent was obtained prior to participation in the experiment. The ability of schizophrenia patients to give informed consent was established using the guidelines of the Canadian Psychiatric Association. The ethics committees of the Fernand-Seguin Research Center of the Louis-H Lafontaine Hospital and the Regroupement Neuroimagerie Québec approved the study.

### 2.2. Procedure

#### 2.2.1. General Protocol

Each woman was scanned twice approximately two weeks apart (±2 days) to examine the cerebral activations associated with the processing of emotional material at two different phases of the menstrual cycle. Prior to each scan, participants were asked about the history of their menstrual cycle. However, due to the high rate of unreliability in the reports, especially in patients who had difficulty keeping track of the first day of their last cycle, we calculated the estradiol to progesterone ratio (E : P ratio) to better differentiate between the follicular and luteal phases of the cycle. Progesterone is significantly higher during the luteal phase of the cycle, while estradiol is the main hormone controlling the follicular phase. Thus, out of the two blood samples taken in the span of two weeks, the one with the significantly higher E : P ratio was indicative of the follicular phase while the blood sample with the lower E : P ratio was indicative of the luteal phase. Women, whose E : P ratio was not significantly different between the two fMRI sessions, were excluded from the final analyses. Moreover, the hormone levels of a few women during the second scan could not be ascertained due to laboratory errors; as a result these women were also excluded from the final analysis. In the end, a total of 15 healthy women and 17 schizophrenia women were included in the analysis.

#### 2.2.2. Hormonal Assays

A blood sample of 10 mL was taken approximately 30 minutes prior to each scanning session to evaluate the levels of estradiol and progesterone in all participants. The sample was immediately centrifuged and the serum separated. The samples were stored (−40°C) and later transported and analyzed at the laboratory of Maisonneuve-Rosemont Hospital. Serum levels of estradiol and progesterone were determined using the automated chemiluminescence assay system (SYNCHRON LX i 725, Beckman Coulter, USA). For estradiol the analytical sensitivity was 20 pg/mL (73 pmol/L) and dynamic range 20–4800 pg/mL (73–17621 pmol/L). For progesterone the analytical sensitivity was 0.08 ng/mL and dynamic range: 0.08–40.0 ng/mL.

#### 2.2.3. Emotion-Processing Task

While in the fMRI scanner, participants passively viewed 48.5-second blocks of emotionally positive, negative, and neutral pictures. The stimuli were selected from the International Affective Picture System (IAPS) [41] based on normative valence and arousal ratings and were matched for content (e.g., people, animals, and landscapes). Each image category was presented in separate blocks, and there were 16-second periods of rest separating the blocks from one another. Each type of block contained 10 images and was repeated 4 times. Each picture appeared for 3000 ms followed by a blank screen with a fixation point for an average of 1.75 s (ranging from 1 to 2.5 s and giving an average interstimulus interval (ISI) of 4.75 s). As a means to ensure that participants were attentive to the presented images during this emotion task, they were asked to indicate with the press of a button whenever they saw a person or part of a person in the picture. To evaluate the participants subjective emotional responses to the presented images, immediately at the end of the first fMRI session, participants were represented with the images of each block and were asked to rate the block of images as whole on a scale ranging from 0 (absence of any emotional reaction) to 8 (strongest emotion ever felt in one's lifetime) the intensity of experienced emotion for each block of stimuli.

#### 2.2.4. Functional MRI Data Acquisition

Blood oxygenated dependent level (BOLD) signals were recorded using a single-shot, gradient-recalled echo-planar imaging sequence (repetition time (TR) = 3000 ms, echo time (TE) = 30 ms, flip angle = 90 degrees, matrix 64 × 64 voxels) on a MRI Siemens TRIO system at 3.0 Tesla, which is operational at the Functional Neuroimaging Unit at the University of Montreal Geriatric Institute. The functional volumes were then registered to individual high-resolution coplanar anatomical images taken during the same scanning session (three-dimensional, spoiled gradient echo sequence; 28 slices, slice thickness = 5 mm, TR = 22 ms, TE = 4 ms, flip angle = 30*″*; matrix 256 × 256 voxels) to better identify activated structures. 

### 2.3. Data Analyses

The demographic data (for the entire sample; Table 1), hormonal levels (for the entire sample and for the subgroup of women tested during two phases of their menstrual cycle; Table 2), as well as subjective ratings of presented stimuli (obtained during the first scan only; Table 3) were analyzed with the Statistical Package for the Social Sciences (SPSS), version 15.0.

The fMRI data were analyzed using statistical parametric mapping software (SPM5; Wellcome Department of Cognitive Neurology, London, UK) according to the methods outlined by Friston [42]. Functional images were realigned to the mean volume of each session to correct for artifacts due to subject motion, were spatially normalized into the standardized brain template (voxel size: 3.5 mm × 3.5 mm × 3.5 mm), and were spatially smoothed with a three-dimensional isotropic Gaussian kernel (12 mm FWHM) to improve the signal-to-noise ratio.

Statistical analyses were carried out using a standard peak-detection approach and the general linear model implemented in SPM5 to identify the dynamic cerebral changes associated with the processing of emotional images. First, fMRI data of each participant were analyzed using a fixed-effects model to investigate individual brain activation maps and to contrast the brain activity associated with different contrasts (i.e., negative versus neutral and positive versus neutral). The fixed-effects analysis produced individual contrast images that were then used as raw data for the implementation of a random-effects model to investigate the pattern of activations during the emotional contrasts in healthy and schizophrenia women. One-sample *t*-tests were implemented to subtract brain activity associated with neutral from that associated with emotional stimuli (emotional minus neutral) for each group. Considering the lack of studies investigating the neural correlates of emotion processing in schizophrenia and healthy women at different phases of the menstrual cycle, we performed an exploratory analysis for the entire brain volume. We also examined any potential differences between groups using a two-sample *t*-test. The threshold level for the statistical significance was set at a *P* = 0.001 uncorrected for multiple comparisons.

In addition to the whole-brain exploratory analysis, the bilateral hippocampus, amygdale, and medial prefrontal cortex were selected as *a priori* regions of interests (ROI) based on existing functional neuroimaging studies of emotion processing (for reviews see: [43–46]). The centers for each of our *a priori* ROIs were produced using the Mask for ROI Analyses software (MARINA) [47]. This software provides 3D masks based on the Automated Anatomical Labelling (AAL) [48]. AAL uses the anatomical boundaries of each region using the MNI template as a reference. A search sphere with a radius of 16 mm was applied to the centre of each ROI using the small volume correction function in SPM5 except for the amygdala in which a sphere of 8 mm was used. The AAL tool in SPM provides the anatomic labelling of each activation peak within the ROI. For the priori search, a probability threshold for multiple comparisons with a corrected *P* < 0.05 and a *z*-score 1.67 was used [49]. Effects at each voxel of the brain were estimated using the general linear model and voxel values for the contrasts of interest-generated statistical parametric maps of the *t* statistic (SPM *t*) that were subsequently transformed to the unit normal distribution (SPM *Z*).

Potential relationships between hormone levels and brain function in healthy and schizophrenia women were investigated using the second-level regression analyses in SPM5. Levels of estrogen and progesterone were entered as covariates of interest. These were correlated first with brain function during positive emotion processing and then with the cerebral activations associated with negative emotion processing for healthy and schizophrenia women separately. To increase the statistical power, the brain function associated with the first fMRI scan of all the women who participated in this study (i.e., 21 patients and 23 controls) was included in the regression analysis (regions were considered significant at *P* < 0.001 uncorrected for multiple comparisons). Where significant relationships were found, data were extracted for each cluster of interest (at the maximum voxel) and entered into SPSS to plot the data and to determine correlation coefficients between the hormone scores and the degree of activation.

## 3. Results

### 3.1. Hormonal Levels and Subjective Ratings of Emotional Stimuli

There were no statistically significant between-group differences in the levels of estrogen or progesterone (please refer to Table 1 and Table 2 for details). Nevertheless, two statistical trends of lowered hormonal levels in the subgroup of SZ-W tested during two phases of their menstrual cycle were noted: estrogen during the follicular phase (*P* = 0.15) and progesterone during the luteal phase (*P* = 0.13).

There were no statically significant between-group differences in subjective ratings of the stimuli (negative, positive, or neutral) during the follicular phase (please refer to Table 3). In comparison, during the luteal phase control women rated negative (but not positive or neutral) stimuli as more emotional than schizophrenia women did (*P* = 0.016).

### 3.2. Functional MRI Data

#### 3.2.1. Negative Emotion Processing

**Figure 1:**
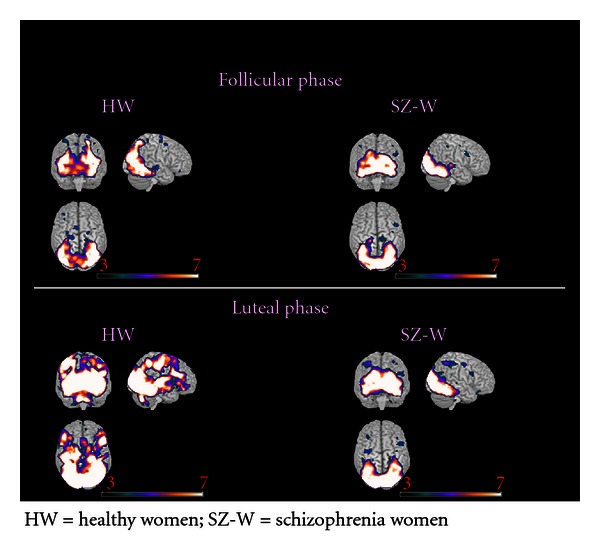
Brain activations during processing of negative emotions.

HW exhibited overall more activations during the luteal than the follicular phase (please refer to Tables 4 and 5, as well as Figure 1). During both menstrual cycle phases, HW activated significantly (among other structures summarized in Tables 4 and 5) bilateral middle occipital cortex, fusiform gyrus, superior frontal and inferior orbitofrontal cortex (OFC), supplementary motor area (SMA), inferior and superior temporal cortex, cerebellum, and hippocampus. These activations were overall more intense and more extensive during the luteal than the follicular phase and the bilateral thalamus, insula and amygdale, were significantly activated during the luteal phase only. 

In SZ-W the activations were comparable in the two phases and included bilateral middle occipital cortex, fusiform gyrus, inferior frontal cortex, SMA, portions of temporal and parietal cortex, cerebellum, and thalamus (for details refer to Tables 4 and 5 and Figure 1).

Of particular interest were the direct comparisons between HW and SZ-W. Thus, the two-sample *t*-tests revealed no significant between-group differences during the follicular phase but did show significantly more activations in HW than in SZ-W during the luteal phase in the bilateral thalamus and inferior frontal cortex, left Heschl gyrus and insula, right superior, and inferior temporal cortex, as well as the hippocampus and middle OFC (for details refer to Tables 4 and 5).

#### 3.2.2. Positive Emotion Processing

**Figure 2:**
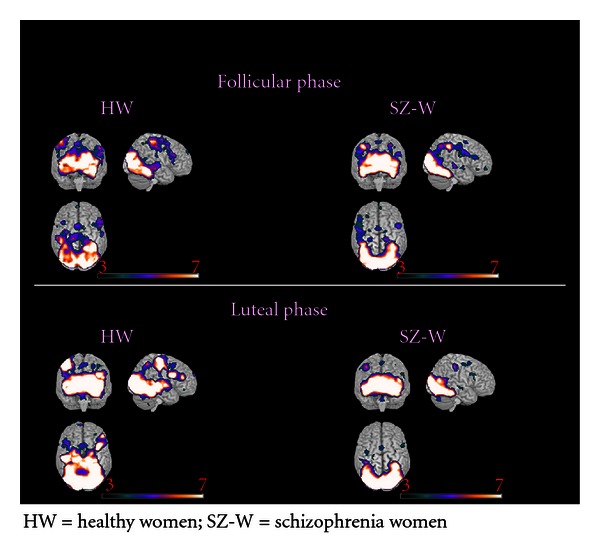
Brain activations during processing of positive emotions.

HW activated similar areas during positive emotion processing in both follicular and luteal phase. These included bilateral occipital cortex, fusiform gyrus, inferior and middle PFC, SMA, inferior temporal and superior parietal cortex, as well as thalamus, hippocampus and cerebellum (for details refer to Tables 6 and 7, as well as Figure 2).

 SZ-W also showed comparable activations during both phases of the menstrual cycle, which included bilateral occipital cortex, fusiform gyrus, thalamus, inferior and OFC, SMA, portions of the parietal cortex, and cerebellum (Tables 6 and 7, as well as Figure 2).

The direct between-group comparisons with the two-samaple *t*-tests revealed significantly more activations in HW than in SZ-W during the follicular phase in the bilateral cerebellum and left calcarine cortex, and during the luteal phase in the right posterior cingulate, precuneus, and left calcarine cortex.

Thus while overall we have observed an interaction between group and menstrual cycle phase during processing of negative emotions, the analysis of positive emotion processing data revealed a significant effect of the group but no interaction.

#### 3.2.3. Hormonal Correlations with Cerebral Activations during Negative Emotion Processing

**Estradiol**

In HW there were positive correlations with activations in the right cerebellum and negative correlations in the left superior frontal cortex, while in SZ-W there were only positive correlations with the left parahippocampal gyrus (please refer to Table 8 for details).

**Progesterone**

There were no significant correlations with progesterone.

#### 3.2.4. Hormonal Correlations with Cerebral Activations during Positive Emotion Processing

**Estradiol**

In HW there were negative correlations with activations in the left amygdala, right inferior frontal, and left inferior parietal cortex, as well as the posterior cingulate. In contrast, in SZ-W there were positive correlations in the right inferior parietal and right cerebellum.

**Progesterone**

In HW there were negative correlations in the left superior temporal and the right superior medial frontal cortex, while there were no significant correlations in SZ-W (please refer to Table 8 for details).

## 4. Discussion

This is the first study demonstrating that atypical neural activations associated with emotion processing in women diagnosed with schizophrenia depend on the menstrual cycle phase and on the affective valence of presented stimuli. During exposure to negatively charged images, we observed an interesting interaction between the diagnostic group (schizophrenia patients versus healthy controls) and phase of the menstrual cycle (follicular versus luteal). Specifically, patients showed relatively less activations than controls during the luteal phase, but no between-group differences were observed during the follicular phase. This effect was apparent due to greater activations during the luteal relative to the follicular phase in healthy women, but lack of increased reactivity to aversive information in women with schizophrenia. In other words, patients appeared more “stable” by exhibiting comparable activations during both phases of their menstrual cycle. In contrast, exposure to positively valenced material produced no significant interaction but did reveal a main effect of group. Thus, in this condition there were no within-group differences between activations during follicular or luteal phase, but schizophrenia patients exhibited less activations than healthy controls during both phases of the menstrual cycle.

 The relative deficit in brain activations, while viewing negatively charged images by schizophrenia patients during the luteal phase, was evident in several limbic and corticolimbic structures (e.g., thalamus, hippocampus, insula, and middle OFC) previously implicated in the processing of affective stimuli [43–46]. These findings are consistent with numerous neuroimaging studies, which showed diminished activations in patients relative to controls during performance of diverse emotional tasks (e.g., [9–12]). However, examination of brain activations in female patients during two stages of their menstrual cycle in the present study revealed that the previously reported “deficit” in processing of negatively valenced material by schizophrenia patients may be restricted to the luteal phase and be absent in the follicular phase.

 Studies of negative affect (anger, fear, and anxiety) often focus on the medial temporal lobe structures; amygdala and hippocampus, both of which have shown deficient activations in schizophrenia patients (e.g., [3, 50, 51]). Indeed, in the present study we have observed that only healthy women exhibited significant activations in the bilateral amygdala and hippocampus during exposure to the unpleasant images in the luteal phase. In comparison, during the follicular phase these structures were not significantly activated in either group, emphasizing again a very specific nature of the observed “deficit” in female patients.

 The between-group differences in brain activations during the positive condition were more restricted than what we have observed during exposure to the negative stimuli in the luteal phase. Thus, in the follicular phase there was less activation in the circumscribed areas of the cerebellum, superior temporal, and calcarine cortex, while in the luteal phase there was less activation in the precuneus, posterior cingulated, and calcarine cortex, in schizophrenia women relative to healthy controls. Although these regions are not considered to be the primary “emotional zones,” they have been activated during emotional tasks. For example, despite the fact that the calcarine fissure's main function is the processing of visual information (this is where the primary visual cortex is concentrated), it has also been shown to respond to emotionally arousing stimuli more than to neutral stimuli even with matched visual complexity [52, 53]. In a similar vein, the cerebellum has been traditionally associated with the motor coordination, but there are numerous studies implicating it in complex cognitive and emotional processing [54], as well as in the pathophysiology of schizophrenia [55, 56].

 Because the principal difference between the two phases of the menstrual cycle is hormonal, the main variables to be taken into consideration while accounting for the observed between-group differences in the pattern of brain activations are the levels of the ovarian hormones in patients relative to controls. However, the levels of estradiol were not significantly different between the two groups of women, and the trend toward a diminished level in patients was present in the follicular (when activations were comparable between the two groups) and not the luteal phase. In contrast, there were significant between-group differences in the progesterone level, which showed a diminished level especially in the luteal phase. Thus, lowered levels of progesterone in patients could partly explain the fMRI data (obtained during processing of negative stimuli), and the results are consistent with some previous studies in healthy women (discussed further below). Moreover, the subjective rating data are consistent with functional neuroimaging findings. Specifically, while there were no significant differences in subjective ratings during the follicular phase, during the luteal phase female patients reported perceiving the negative stimuli as less emotional than healthy controls. It should be noted, however, that the subjective rating data were collected only during the first scanning session.

 The findings in control women are consistent with several previous reports. For example, response of the OFC has been shown to vary during exposure to negatively valenced words; the activity was increased premenstrually during the luteal phase (when estrogen levels drop), and increased postmenstrually during the early follicular phase (when estrogen levels rise) [32]. In a different study, in addition to changes in the OFC, significantly greater responses to negatively valenced and highly arousing stimuli were found during the early follicular compared with late follicular phase (when estrogen levels are at its maximum during ovulation) in the amygdala, hippocampus, anterior cingulated, and hypothalamus [31]. Similarly, a more recent study has shown greater activity in the amygdala and OFC while passively viewing blocks of faces morphing dynamically from a neutral into either angry, happy, or fearful expression, during the late luteal relative to the late follicular phase (interestingly, stress induction had opposite effects in the two cycle phases) [30]. In another recent study, increased neural responses to negative images have been observed during the luteal as compared to the follicular phase, in the hippocampus and amygdala [24]. Because (similarly to our study) the differences between estradiol levels were not significantly different between the two phases, the effect was attributed mainly to different levels of progesterone (higher in the luteal phase). Overall, these studies imply that enhanced levels of estradiol are associated with the attenuated cerebral reactivity, whereas progesterone appears to enhance the neural responsiveness to negative emotional stimuli. The results obtained in healthy women in the present study are consistent and extend these previous reports. Thus, we have also observed enhanced cerebral activations to negatively valenced stimuli during luteal relative to follicular stage, but interestingly during processing of positive images there were no phase-dependent differences. This suggest that the “reactivity” is specific for aversive material, at least in healthy premenopausal women as we did not observe this effect in female schizophrenia patients.

 Because ovarian hormones have been implicated in brain function associated with processing of emotional material [24, 30–32], in addition to comparing fMRI results in the two phases of the menstrual cycle, we also performed correlational analyses between cerebral activations and levels of both estradiol and progesterone in all participants who completed the first scanning session. The overall findings were rather intriguing as majority of correlations were in the opposite direction in the two diagnostic groups. Thus, elevated levels of estradiol in control women were associated with decreased activation in the left superior frontal cortex during processing of unpleasant stimuli, and in the right inferior frontal, left inferior parietal, and posterior cingulate cortex during positive condition. In contrast, in women with schizophrenia levels of estradiol were related to enhanced activations in the left parahippocampal gyrus during processing of negative images, and right inferior parietal and right cerebellum during positive emotions. In other words, the higher the levels of estradiol in healthy women, the less neural sensitivity to the emotional material, while in the group of patients this relationship was reversed.

 This finding is puzzling because there were no significant differences in the overall level of estradiol between the groups. Nevertheless, it is possible that even with the comparable levels of ovarian hormones, the interaction with illness processes may alter what is considered the optimal levels for patients and how these hormones interact with brain function and emotion processing. The ovarian hormones, particularly estradiol, have been implicated in the pathophysiology of schizophrenia for the past few decades. For example, symptoms have been reported to fluctuate across the menstrual cycle in women with schizophrenia such that there is clinical deterioration during times associated with low estrogen (e.g., premenstrual/late luteal phase) and amelioration during high estrogen (late follicular/ovulatory phase) [57, 58]. Also, during pregnancy, when estrogens (and progesterone) levels are high, low rates of relapse have been observed in women with schizophrenia [59], and there is an increase in relapse postpartum when estrogens levels drop abruptly [60]. In terms of the altered levels of sex steroid hormones, the evidence is more equivocal. Some studies, which found diminished levels of estrogens in schizophrenia women, attributed the effect to the antipsychotic-induced hyperprolactinaemia, mediated by hypothalamic-pituitary-ovarian feedback mechanisms, while others have argued that hypoestrogenism in schizophrenia women occurs independently of antipsychotic use (e.g., [33, 34]). In the present study we did not see any significant differences, but there were some statistical trends towards a diminished level of estradiol in patients relative to controls.

 Overall, present findings augment our knowledge concerning neural correlates of affect in psychosis. Furthermore, they emphasize the importance of studying the sexes separately and taking the hormonal status of female patients into consideration when investigating processing of emotional material.

## Figures and Tables

**Table 1:** Demographic, clinical, and hormonal characteristics of control and schizophrenia women.

	Control women		Schizophrenia women		
	(*N* = 25)		(*N* = 22)		*P*
	M	SD	M	SD	
*Demographic*					
Age (years)	29.28	9.27	32.86	6.56	0.138
Parental SES	2.08	1.10	2.55	1.05	0.145
*Clinical*					
PANSS positive	—	—	19.32	7.82	—
PANSS negative	—	—	20.14	8.69	—
PANSS general	—	—	42.27	12.71	—
Medication (mg/day)	—	—	496.61	267.94	—
*Hormonal*					
Estrogen (pmol/L)	256.42	265.38	214.90	150.80	0.525
Progesterone (nmol/L)	12.93	19.37	8.66	10.98	0.371

M: mean; SD: standard deviation; SES: socioeconomic status; Medication: equivalence in chlorpromazine.

**Table 2:** Hormone levels in the subgroup of women tested during both phases of the menstrual cycle.

	Follicular phase				*P*	Luteal phase				*P*
	Control women		Schizophrenia women			Control women		Schizophrenia women		
	(*N* = 15)		(*N* = 17)			(*N* = 15)		(*N* = 17)		
	M	SD	M	SD		M	SD	M	SD	
Estrogen	450.13	587.63	228.45	162.58	0.15	256.00	236.65	197.34	130.67	0.39
Progesterone	2.11	2.74	1.45	1.12	0.36	22.99	26.42	12.08	10.44	0.13

M: mean; SD: standard deviation; Plasmatic estrogen is in pmol/L; plasmatic progesterone is in nmol/L.

**Table 3:** Subjective ratings of control and schizophrenia women divided into the follicular and luteal phases.

	Follicular phase				*P*	Luteal phase				*P*
	Control women		Schizophrenia women			Control women		Schizophrenia women		
	(*N* = 9)		(*N* = 11)			(*N* = 9)		(*N* = 10)		
	M	SD	M	SD		M	SD	M	SD	
SR negative	5.56	0.54	5.43	1.22	0.782	6.06	0.60	4.83	1.25	0.016*
SR positive	5.14	1.02	4.66	0.96	0.296	5.00	0.86	4.85	1.47	0.793
SR neutral	1.06	0.93	1.73	0.96	0.131	1.08	0.75	1.60	1.46	0.354

M: mean; SD: standard deviation; SR: subjective ratings. *≤0.05.

**Table 4:** Cerebral activations during processing of negative emotions in control and schizophrenia women during the follicular phase.

Anatomical area	L/R	Control women						Schizophrenia women					
		BA	MNI coordinates			*Z*-score	Voxels	BA	MNI coordinates			*Z*-score	Voxels
			*x*	*y*	*z*				*x*	*y*	*z*		
Fusiform	R	20	42	−35	−21	4.73	4196	19	35	−70	−18	5.95	3834
	L	19	−28	−70	−14	4.55	*	19	−24	−80	−18	5.73	*
Calcarine	R							18	4	−88	−10	6.17	*
Middle occipital	R	18	35	−91	−18	6.07	*	19	32	−91	10	5.26	*
	L	18	−35	−88	10	5.41	*	19	−35	−80	4	4.76	*
Inferior occipital	L	37	−46	−70	−4	6.25	*						
	R							19	46	−80	−7	4.97	*
Superior occipital	R	17	21	−88	7	4.86	*	18	24	−91	18	5.35	*
Inferior temporal	R	37	42	−52	−7	5.46	*						
Hippocampus	L	—	−28	−24	−10	3.51	81						
Anterior temporal pole	L	38	−35	24	−24	3.47	6						
Cerebellum	L	—	−10	−74	−21	4.45	4196	—	−38	−52	−28	5.63	*
	R	—	32	−56	−28	4.91	*						
Thalamus	R							—	10	−24	−4	3.60	37
Superior parietal	L	7	−24	−63	52	3.50	13						
Inferior parietal								40	−38	−38	46	3.40	11
Precentral	R	9	52	4	38	3.20	5	9	49	4	38	3.19	37
	L	6	−28	−10	56	3.48	19						
Middle frontal	R	6	38	0	52	3.26	6						
Superior frontal	R	6	24	−10	74	3.78	18						
Inferior OFC	L	11	−35	38	−18	3.72	7						
Inferior frontal								9	52	10	28	3.60	37
SMA	L	6	−4	4	52	3.79	22	6	0	7	56	3.16	5

L: left; R: right; BA: Brodmann area; SMA: supplementary motor area; OFC: orbitofrontal cortex; *P* uncorrected ≤0.001.

**Table 5:** Cerebral activations during processing of negative emotions in control and schizophrenia women during the luteal phase.

Anatomical area	L/R	Control women						Schizophrenia women					
		BA	MNI coordinates			*Z*-score	Voxels	BA	MNI coordinates			*Z-*score	Voxels
			*x*	*y*	*z*				*x*	*y*	*z*		
Fusiform	R	19	32	−77	−18	6.78	7436	19	38	−70	−18	5.05	4678
	L	37	−38	−46	−24	6.12	*	19	−38	−74	−18	5.62	*
Calcarine	L	17	−7	−98	0	6.28	*	17	−4	−91	−4	5.68	*
Cuneus	R	19	10	−98	21	6.33	*						
Middle occipital	R	19	32	−91	14	7.40	*						
	L	19	−28	−84	21	5.85	*	19	−28	−94	14	6.19	*
Inferior occipital	R							18	42	−84	−7	5.71	*
Inferior temporal	R	19	49	−74	−7	7.43	*	22	42	−60	14	3.54	*
Thalamus	L	—	−21	−24	0	5.85	*						
	R	—	24	−28	7	5.69	*	—	21	−28	4	3.47	7
Amygdala	R	—	28	0	−14	4.32	14						
Insula	L	13	−32	24	0	4.46	36						
Anterior temporal pole	R							38	42	24	−28	3.61	7
Cerebellum	L	–	−38	−74	−21	5.65	7436	—	−21	−32	−46	3.47	6
	R	–	10	−70	−42	5.21	*	—	24	−32	−46	3.41	9
Supramarginal	R	40	46	−32	42	4.74	182						
Inferior parietal	R	7	28	−52	49	4.50	*						
	L							7	−32	−52	52	4.1	119
Postcentral	L							40	−49	−32	49	3.57	*
Superior parietal	L							7	−28	−60	52	3.93	*
Precentral	L	9	−56	10	32	4.67	193						
	R							6	42	−7	49	3.32	6
Inferior frontal	R	9	52	14	24	5.63	222	9	46	10	24	3.63	52
	L	46	−46	28	21	5.08	193						
Middle frontal	L							6	−28	−7	49	3.87	51
Superior medial frontal	L	32	−4	18	42	3.89	70						
Superior frontal	R	6	35	−4	66	4.75	29						
Inferior OFC	R	11	42	35	−14	4.63	40						
	L	47	−46	35	−14	3.91	6	47	−32	28	−21	4.08	18
SMA	R	8	4	14	56	4.23	70						

		Controls greater than patients						Patients greater than controls					

Fusiform	L	20	−38	−14	−24	3.00	1376						
Calcarine	L	18	−4	−102	0	3.34	25						
Cuneus	R	7	14	−77	32	2.99	14						
Heschl	L	13	−32	−32	10	4.00	1376						
Superior temporal	R	22	66	−10	7	3.35	15						
Inferior temporal	R	20	60	−18	−24	3.09	9						
Thalamus	L	—	−24	−24	7	3.60	1376						
	R	—	10	−10	7	3.22	*						
Hippocampus	R	—	14	−38	7	3.80	*						
Insula	L	—	−35	0	10	3.19	*						
Inferior parietal	L	7	−24	−52	52	4.05	90						
	R							40	56	−60	38	3.02	7
Superior parietal	R	7	21	−56	56	3.20	38						
Inferior frontal	L	46	−42	32	24	3.67	117						
	R	45	52	35	7	3.01	6						
Middle OFC	R	11	38	52	−14	3.09	10						

L: left; R: right; BA: Brodmann area; SMA: supplementary motor area; OFC: orbitofrontal cortex; *P* uncorrected ≤0.001.

**Table 6:** Cerebral activations during processing of positive emotions in control and schizophrenia women during the follicular phase.

Anatomical area	L/R	Control women						Schizophrenia women					
		BA	MNI coordinates			*Z*-score	Voxels	BA	MNI coordinates			*Z-*score	Voxels
			*x*	*y*	*z*				*x*	*y*	*z*		
Fusiform	R	19	38	−49	−10	5.64	4702	18	24	−84	−14	5.91	4870
	L	37	−35	−46	−18	4.45	*	37	−38	−60	−18	5.70	*
Middle occipital	R	19	32	−94	14	5.45	*						
	L	18	−21	−94	7	5.29	*	18	−24	−98	7	5.28	*
Superior occipital	L	17	−18	−91	4	5.29	*	18	−10	−102	7	5.68	*
	R	18	14	−94	18	4.98	*	18	18	−94	18	5.42	*
Inferior occipital	L	19	−46	−77	−4	5.29	*	19	−42	−80	−10	5.02	*
	R	19	38	−80	−4	4.93	*	19	35	−66	−10	4.93	*
Calcarine	L	18	−14	−74	4	4.87	*						
	R	18	7	−77	4	4.85	*						
Cuneus	R	18	14	−94	10	5.06	*						
Cerebellum	R	—	18	−49	−18	4.78	*	—	35	−46	−24	5.03	*
Inferior temporal	L	37	−42	−63	−10	4.55	*						
Parahippocampal	R	35	21	−35	−10	4.06	*						
Thalamus	L							—	−21	−28	0	3.82	40
Pallidum	L							—	−21	−7	−7	3.58	13
Postcentral	L	2	−46	−35	60	4.69	433						
Superior parietal	L							7	−28	−60	49	4.20	4870
Inferior parietal	L	2	−56	−24	46	4.13	*						
Supramarginal	R							40	38	−35	42	3.23	8
Precentral	R	8	56	10	42	4.59	302	9	52	4	28	3.97	68
Inferior frontal	R	44	63	18	14	4.27	*						
	L	13	−38	14	18	3.78	26						
Medial OFC	R							11	7	52	−10	3.40	18
Inferior OFC	L							47	−28	28	−14	3.32	15
Superior medial frontal	L	9	−4	56	32	3.37	25						
SMA	L	32	−4	7	52	3.85	114	6	−4	0	56	3.76	41

		Controls greater than patients						Patients greater than controls					

Superior temporal	R	13	46	−46	21	3.29	17						
Cerebellum	L	—	−24	−35	−35	3.19	29						
	R	—	24	−32	−32	3.00	91						
Calcarine	L	30	−10	−66	10	3.17	139						

L: left; R: right; BA: Brodmann area; SMA: supplementary motor area; OFC: orbitofrontal cortex; *P* uncorrected ≤0.001.

**Table 7:** Cerebral activations during processing of positive emotions in control and schizophrenia women during the luteal phase.

Anatomical area	L/R	Control women						Schizophrenia women					
		BA	MNI coordinates			*Z*-score	Voxels	BA	MNI coordinates			*Z*-score	Voxels
			*x*	*y*	*z*				*x*	*y*	*z*		
Fusiform	R	19	32	−52	−10	5.76	8191						
	L							19	−38	−70	−18	6.33	4076
Middle occipital	L	17	−10	−94	0	6.23	*	18	−24	−94	7	5.76	*
Inferior occipital	R	19	42	−80	−4	6.16	*						
	L							18	−49	−80	−7	6.16	*
Hippocampus	R	—	18	−35	0	5.22	*						
Posterior cingulate	R	29	10	−42	7	5.01	*						
Cerebellum	R	—	14	−52	−49	3.49	*	—	4	−74	−38	3.77	4076
Postcentral	L							40	−38	−35	46	4.40	90
Supramarginal	L							3	−56	−24	42	3.30	*
Precentral	L							6	−38	−10	52	3.73	30
	R							6	42	−7	49	3.30	6
Inferior frontal	R	9	38	7	24	5.46	480	9	56	7	28	3.41	34
Medial frontal	L	10	−7	63	24	4.04	35						
Inferior OFC	R	11	28	35	−10	3.48	8						
Superior frontal	R	6	35	−4	66	3.31	7						
SMA	L	32	−7	7	46	3.66	96	6	0	4	60	3.67	33

		Controls greater than patients						Patients greater than controls					

Posterior cingulate	R	29	7	−42	10	3.55	106						
Calcarine	L	30	−21	−52	10	3.41	*						
Precuneus	R	30	21	−46	7	3.36	*						

L: left; R: right; BA: Brodmann area; SMA: supplementary motor area; OFC: orbitofrontal cortex; *P* uncorrected ≤0.001.

**Table 8:** Correlations between hormone levels and cerebral activations during processing of emotion (negative and positive) in control and schizophrenia women.

Correlation	Group	Anatomical area	L/R	BA	MNI coordinates			*Z*-score	Voxels
					*x*	*y*	*z*		
Estrogen									

*Negative emotion*									
+	CW	Cerebellum	R	—	24	−60	−42	3.14	13
−		Superior frontal	L	6	−21	21	60	3.18	31
+	SZW	Parahippocampal	L	28	−21	−21	−21	3.68	21

*Positive emotion*									
−	CW	Inferior parietal	L	40	−38	−32	38	3.23	20
		Amygdala	L	—	−24	−4	−14	3.14	24
		Inferior frontal	R	13	38	10	24	3.10	9
		Posterior cingulate	L	29	−4	−42	10	3.01	46
+	SZW	Inferior parietal	R	40	35	−42	52	3.09	17
		Cerebellum	R	—	18	−32	−21	2.98	29

Progesterone									

*Positive emotion*									
−	HW	Anterior temporal pole	L	38	−56	14	−18	3.36	13
		Superior medial frontal	R	10	10	60	14	3.03	18

L: left; R: right; BA: Brodmann area; (−): negative correlation; (+): positive correlation; CW: control women; SZW: schizophrenia women; *P* uncorrected ≤0.001.
